# VL-HIV co-infection with *Leishmania* containing skin lesions resembling para-kala-azar dermal leishmaniasis

**DOI:** 10.1371/journal.pntd.0012438

**Published:** 2024-08-26

**Authors:** Natália O. Alves, Jéssica A. Oshiro, Yunna C. Silva, Gabriela C. Pacher, Aline E. Casaril, Yasmin S. Rizk, Silvia N. O. Uehara, Anamaria M. M. Paniago, Isadora L. X. Andrade, Carla C. P. Arruda, Alessandra G. Oliveira

**Affiliations:** 1 Laboratory of Human Parasitology, Institute of Biosciences, Federal University of Mato Grosso do Sul, Campo Grande, Brazil; 2 Graduate Program in Infectious and Parasitic Diseases, Faculty of Medicine, Federal University of Mato Grosso do Sul, Campo Grande, Brazil; 3 Faculty of Medicine, Federal University of Mato Grosso do Sul, Campo Grande, Brazil; 4 Graduate Program in Pharmaceutical Sciences, Faculty of Pharmaceutical Sciences, Food and Nutrition, Federal University of Mato Grosso do Sul, Campo Grande, Brazil; 5 Maria Aparecida Pedrossian University Hospital—Federal University of Mato Grosso do Sul, Campo Grande, Brazil; Institute of Infectology Emilio Ribas and Instituto de Medicina Tropical, BRAZIL

## Abstract

Leishmaniases are a group of neglected vector-borne infectious diseases that are among the six priority endemic diseases worldwide. Visceral leishmaniasis (VL) is the most severe clinical manifestation, characterized by systemic and chronic visceral involvement and high mortality in immunosuppressed and untreated patients. VL can be complicated into post-kala-azar dermal leishmaniasis (PKDL), and when dermatologic disorders occur simultaneously with active VL, an intermediate clinical form called para-kala-azar dermal leishmaniasis (para-KDL) occurs. This clinical form is of great epidemiological relevance, as humans act as a source of infection for vectorial transmission. In the Americas, Brazil is among the seven countries responsible for more than 90% of VL cases, though reports of PKDL and para-KDL are rare. This paper presents three cases of VL-HIV co-infection with *Leishmania*-containing skin lesions resembling para-kala-azar dermal leishmaniasis. The cases were investigated by the team from the Infectious Diseases Department of University Hospital (HUMAP/UFMS) in Mato Grosso do Sul, Brazil. The three patients exhibited skin lesions where amastigote forms of *L*. *(L*.*) infantum* were identified. All cases exhibited similar clinical manifestations of para-KDL, including fever, hepatosplenomegaly, pancytopenia, and disseminated skin lesions. The study described the prevalence of comorbidities, the incidence of VL relapse, and the therapeutic regimen in relation to the outcomes. The study underscores the importance of follow-up and secondary prophylaxis in patients with VL, which are essential for the efficacy of the treatment. Furthermore, the study provides insight into the potential epidemiological profile of para-KDL cases in Brazil, which contributes to the development of more efficient clinical management strategies for patients.

## Author summary

Visceral leishmaniasis (VL) is a neglected vector-borne infectious disease with a systemic and chronic involvement that is lethal when untreated. Para-kala-azar dermal leishmaniasis (para-KDL) is a complication of VL, in which the patient presents with dermatological and visceral manifestations simultaneously. Para-KDL cases are of significant epidemiological importance, as humans act as a source of infection in parasite transmission. In the Americas, Brazil accounts for over 90% of VL cases. Nevertheless, reports of para-KDL remain scarce. This paper presents three cases of VL-HIV co-infection with *Leishmania*-containing skin lesions resembling para-kala-azar dermal leishmaniasis. The three cases exhibit similarities in terms of signs and symptoms, diagnostic criteria for AIDS, and the presence of active VL with skin lesions. The study contributes to the understanding of the epidemiological profile of para-PKDL cases in Brazil. It highlights the significance of multidisciplinary follow-up and secondary prophylaxis for patients with VL and emphasizes the need for more effective clinical management strategies for these patients.

## Introduction

Leishmaniases, a group of vector-borne infectious diseases caused by several species of *Leishmania* protozoan parasites, are considered neglected tropical diseases by the World Health Organization (WHO). They are included among the six priority endemic diseases worldwide, given that they occur in countries with vulnerable populations. In the Americas, these diseases represent a significant public health concern due to their high incidence, extensive geographic distribution, and high morbidity and mortality rates [1,2].

The clinical manifestations of leishmaniasis are dependent upon the causative species and the patient’s immune response and can be classified as cutaneous leishmaniasis (CL), mucocutaneous leishmaniasis (MCL), and visceral leishmaniasis (VL) [1]. Visceral leishmaniasis (VL), also known as kala-azar, is the most severe clinical manifestation, with a systemic and chronic involvement of the viscera. It is important to note that VL is a highly lethal disease in untreated and immunosuppressed individuals, particularly in patients with *Leishmania*-HIV co-infection [3,4].

In the 1990s, the concurrence of HIV and leishmaniasis was first observed in countries along the Mediterranean basin [5]. Currently, there is a high frequency of *Leishmania*-HIV co-infection in Ethiopia and Brazil [6]. HIV-positive patients are particularly vulnerable to VL since this disease contributes to the acceleration of HIV replication and its progression to AIDS. This interaction between the two conditions represents a substantial challenge in regions with a high prevalence of co-infection [7].

Visceral leishmaniasis (VL) can result in the development of post-kala-azar dermal leishmaniasis (PKDL), a dermatological disorder that can lead to stigmatization [8]. The symptoms of PKDL typically manifest between six months and several years after the apparent clinical remission of VL. The presence of macular, papular, or nodular skin lesions is indicative of PKDL, in which *Leishmania* parasites can be visualized upon biopsy [9]. The incidence of PKDL is highest in African and Southeast Asian countries, where *Leishmania donovani* is the primary causative agent. A review of the literature revealed a paucity of documented cases from other regions [3].

In the event of dermatological disorders occurring concurrently with active VL, they give rise to an intermediate clinical form, designated Para-Kala-azar Dermal Leishmaniasis (para-KDL) [10]. The clinical manifestations of para-KDL are characterized by the development of skin rashes that may originate around the mouth and subsequently spread to other parts of the body. A study conducted in Bangladesh observed a combination of the clinical-pathological conditions of VL and PKDL in patients with para-KDL, who presented with a history of fever for weeks or months and constitutional symptoms such as weight loss, weakness, hepatosplenomegaly, anemia, and macular or mixed skin lesions (macular, papular, and nodular) [11]. Phenotypes may include the hypopigmented flat (macular) rash, which may be accompanied by some mildly raised (maculopapular) or raised (nodular) rash [9].

Brazil is one of seven countries with the highest number of cases, accounting for over 90% of VL cases [3]. However, reports of PKDL and para-KDL are uncommon [8,12–15]. Mato Grosso do Sul state is considered an endemic region for VL, with 152 new cases and 19 deaths registered in 2022, representing a 50% increase compared to the previous year [16]. Currently, there are no reports of para-KDL in the region.

This paper presents three cases of immunocompromised patients with active VL and *L*. *(L*.*) infantum*-HIV coinfection, which exhibit similar characteristics to those observed in para-KDL.

## Methods

### Ethics statement

The study received approval from the Human Research Ethics Committee of the Federal University of Mato Grosso do Sul (CEP/UFMS- 4.628.192), as well as the Human Research Ethics Committee of the Maria Aparecida Pedrossian University Hospital (HUMAP/UFMS), under protocol 6/2021/SGPIT/GEP/HUMAP-UFMS-EBSERH. Data contain potential information. Written informed consent was obtained from patients.

### The study participants

This paper presents three cases of patients treated at the Maria Aparecida Pedrossian University Hospital (HUMAP/UFMS) in Campo Grande, Mato Grosso do Sul, Brazil. The patients exhibited similar clinical characteristics, prompting further investigation by the medical and research team. The hospital conducted routine examinations of suspected cases of VL and collected biological material for direct parasitological diagnosis.

The Human Parasitology Teaching and Research Laboratory of the Biosciences Institute at the Federal University of Mato Grosso do Sul (LPH/INBIO/UFMS) collaborated in the analysis of collected materials, including bone marrow aspirate (BMA), lesion edge biopsy, and slides. The materials were subjected to indirect parasitological examination and molecular diagnosis.

### Indirect parasitological examination

The bone marrow aspirate (BMA) samples were inoculated into NNN biphasic medium with liquid phase of Schneider’s insect medium (Sigma-Aldrich, SP/Brazil) supplemented with 20% fetal bovine serum (Sigma-Aldrich, SP/Brazil), 10,000 U.mL-1 penicillin, 10 mg.mL-1 streptomycin (Sigma-Aldrich, SP/Brazil), and 0.5 mL of male urine. The cultures were incubated at 26°C and observed for the presence of promastigotes, mobile flagellated forms, every seven days for six weeks [17,18].

For samples of biopsy material from the lesion edge, the biological material was washed three times in Schneider’s insect medium (Sigma-Aldrich, SP/Brazil) supplemented as described above [17]. The material was macerated and inoculated under uniform conditions to prevent contamination by bacteria and fungi.

### Molecular diagnosis and species characterization

BMA, cutaneous lesion samples, and slide scrapings were used for molecular diagnosis. These materials were fixed, stained, and subjected to DNA extraction. The PureLink Genomic DNA kit (Invitrogen, Waltham, United States) was used to extract DNA from the BMA and biopsy materials, following the manufacturer’s instructions. Lesion edge biopsy slides were scraped with a scalpel, and DNA extraction followed the instructions for the NucleoSpin Tissue kit (Macherey-Nagel, Düren, Germany).

The diagnosis was made using polymerase chain reaction (PCR) to identify a 300 base pair (bp) region of the ribosomal internal transcribed spacer 1 (ITS1). The primers LITSR (5’ CTGGATCATTTTCCGATG 3’) and L5.8S (5’ TGATACCACTTATCGCACTT 3’) were utilized. For the polymerase chain reaction (PCR), a solution of 8 μL ultrapure water, 10 μL of 2x Conventional MasterMix Buffer (QuatroG Biotecnologia, Porto Alegre, Brazil), 1 μL of each oligonucleotide (10 pm/μL), and 5 μL of extracted DNA was prepared in a final volume of 25 μL. The thermal gradient profile described by Schönian (2003) was followed [19]. The PCR products were subjected to electrophoresis on a 1.5% agarose gel, and the resulting bands were visualised using ultraviolet light.

The *Leishmania* species was identified through the use of the RFLP (Restriction Fragment Length Polymorphism) technique with PCR products. The products were digested with HaeIII restriction enzymes (isolated from *Haemophilus aegyptius*), which cleave fragments into segments with the 5’… .GG▼CC… .3’ or 3’… .CC Sequence ▲GG… .5’ [1]. The reaction consisted of a final volume of 20 μL, including 15 μL of pure ultra water, 2 μL of 10x buffer, 1 μL of HaeIII enzyme, and 2 μL of PCR DNA. The sample was incubated at 37°C in a water bath for one hour and then for 20 minutes at 80°C. Subsequently, the material underwent electrophoresis in a 2% agarose gel with TBE buffer for two hours, and the bands were observed under ultraviolet light. The expected fragment sizes for identifying *L*. *infantum* are 50 bp and 200 bp, and for *L*. *amazonensis* they are 200 bp and 140 bp [19].

## Results

### Case 1

**Fig 1:**
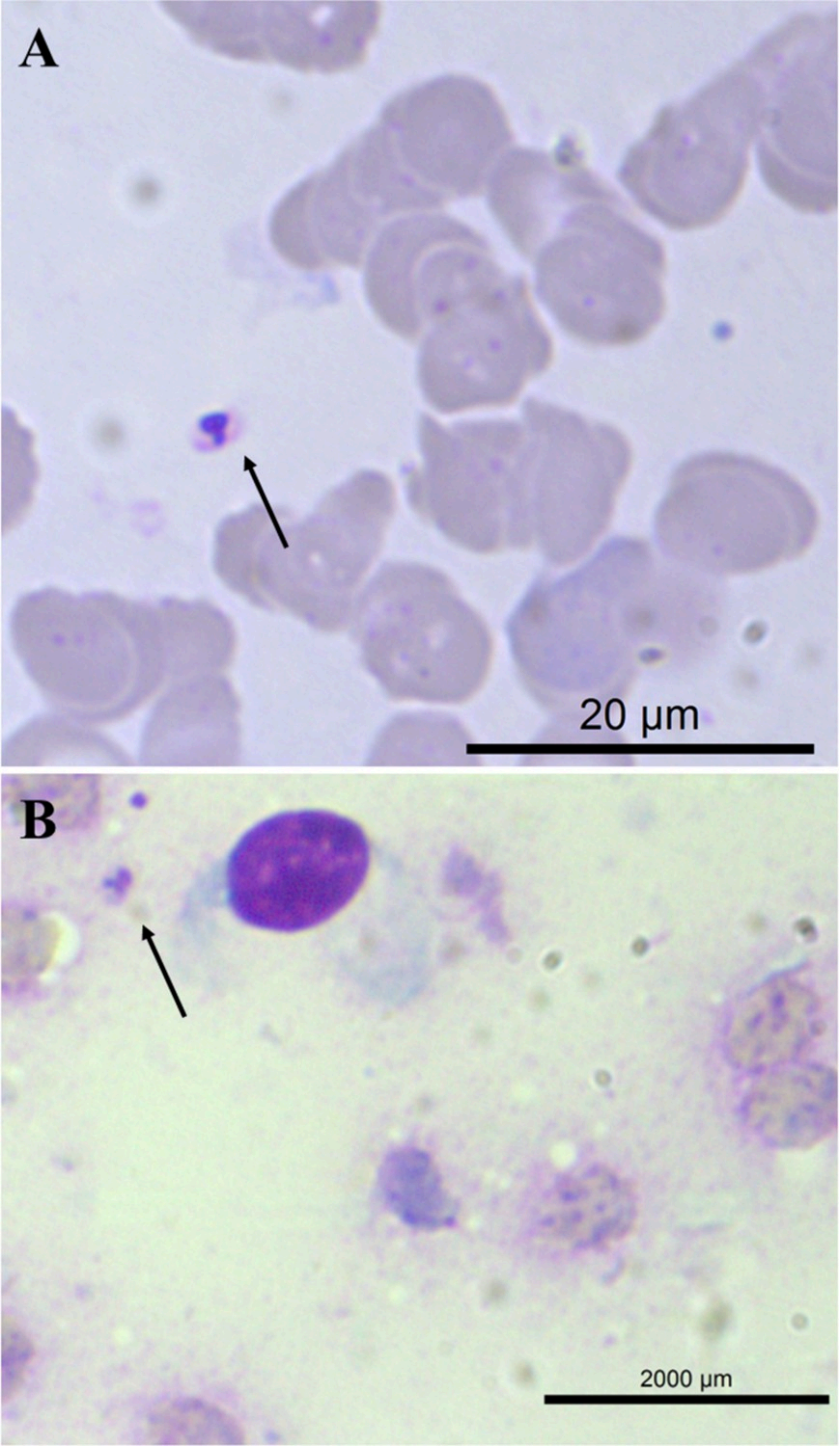
Diagnosis of leishmaniasis (Case 1): direct parasitological methods. **(**A) Amastigote form in bone marrow aspiration (arrow); (B) amastigote form found in skin lesion biopsy (arrow).

In November 2021, a 28-year-old male street vendor from Campo Grande, MS, was referred to Maria Aparecida Pedrossian University Hospital (HUMAP/UFMS) with acquired immunodeficiency syndrome (AIDS) and syphilis. His CD4 count was 16 cells/mm^3^. The patient reported a loss of weight and an itchy rash, accompanied by the appearance of lesions that were pale and ulcerated, particularly in the upper and lower extremities. These lesions had first manifested two months prior. In the preceding two weeks, the patient exhibited symptoms of hyporexia, asthenia, odynophagia, fever (not measured), night sweats, and dry cough. Additionally, he reported an anus nodule that impeded bowel movements and caused bleeding. His medical history included a history of alcoholism, tobacco smoking for 10 years, and the use of illicit drugs, including cocaine, marijuana, and base paste. The patient’s physical examination revealed a poor clinical state, with emaciation, dehydration, and pallor. Additionally, lymph node enlargement was observed in the retroauricular, anterior, and posterior cervical chains. Disseminated nodular lesions were observed predominantly in the face and limbs, in addition to hypochromic lesions in the upper limbs. No palpable visceromegaly or other alterations were detected. During the patient’s hospitalization, amastigote forms of *Leishmania* sp. were observed in the bone marrow aspirate (BMA) and peripheral blood, as well as in the biopsy of skin lesions (Fig 1A and 1B). Additionally, he was diagnosed with pulmonary tuberculosis and esophageal candidiasis. He presented with severe pancytopenia and received a transfusion of three units of packed red blood cells. He commenced treatment for leishmaniasis with 50 mg/day of amphotericin B deoxycholate but was discharged from the hospital after six days without HAART. He declined to continue the treatment regimen despite being informed of its necessity.

**Fig 2:**
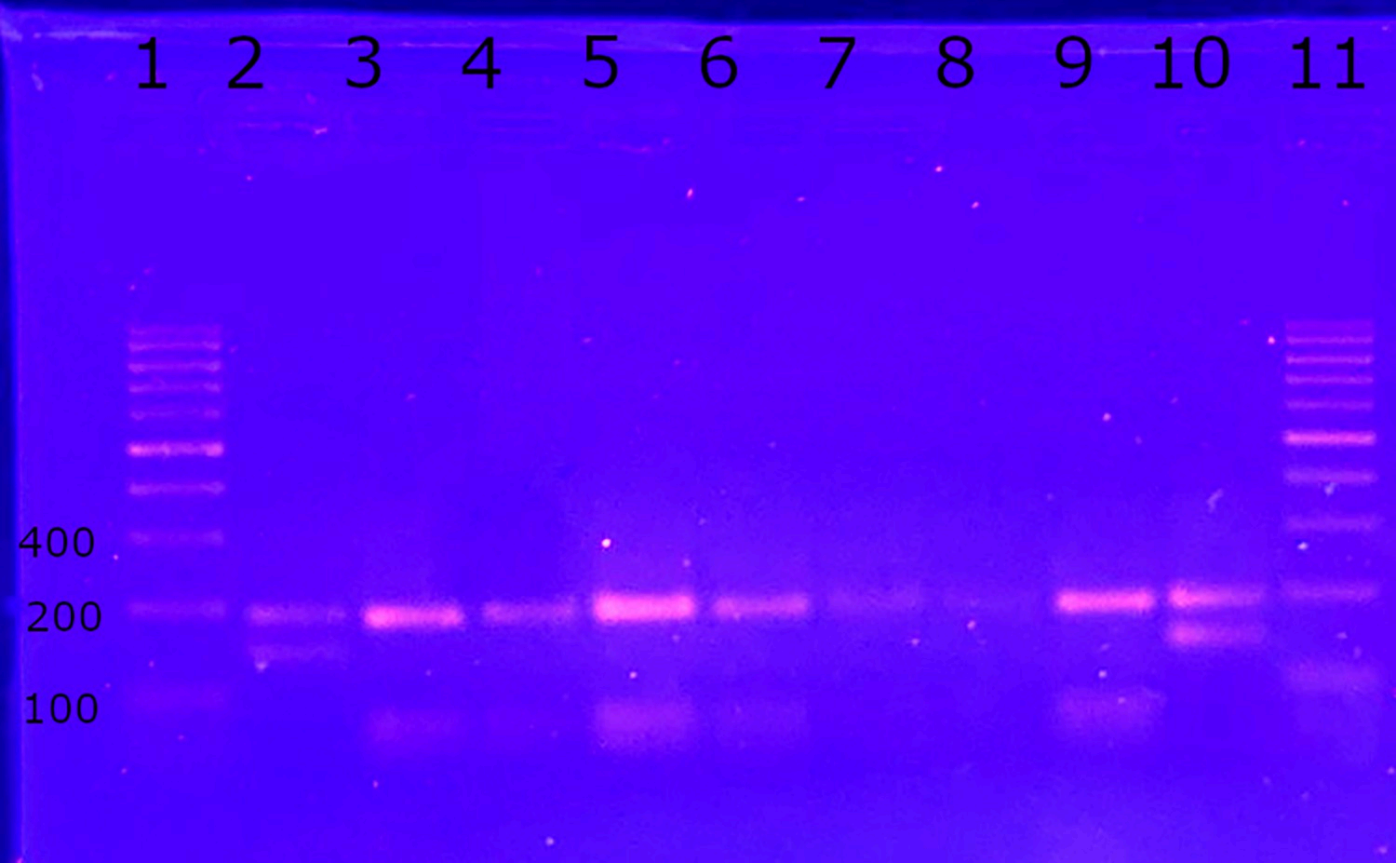
Molecular diagnosis by RFLP. Positive product amplifications underwent digestion with the HaeIII restriction enzyme. 1: Ladder 100 bp; 2: positive control for *Leishmania* (*Leishmania*) *amazonensis* (IFLA/BR/1967/PH8), fragments of approximately 140 and 200 bp. 3: positive control for *Leishmania* (*Leishmania*) *infantum* (MHOM/BR/2022/072) fragments of approximately 50 and 200 bp. 4: Bone marrow aspirate (BMA) sample (Case 1). 5: Skin lesion biopsy sample (Case 1). 6: BMA sample (Case 2). 7: BMA sample (Case 3). 8: Skin lesion biopsy sample (Case 3). 9: positive control for *L*. (*L*.) *infantum* (MHOM/BR/2022/072). 10: positive control for *L*. (*L*.) *amazonensis* (IFLA/BR/1967/PH8). 11: Ladder 100 bp.

In November 2021, samples of BMA and skin biopsy were submitted for an indirect parasitological test (axenic culture) and molecular diagnosis to the Human Parasitology Laboratory of the Biosciences Institute of the Federal University of Mato Grosso do Sul (LPH/INBIO/UFMS). Flagellated parasites were observed in the culture from the first week. A conventional PCR was performed using primers targeting a region of approximately 300 base pairs (bp) of the internal transcribed spacer region of the *Leishmania* ribosomal gene (ITS1), and the parasite DNA was successfully detected in both materials. The RFLP technique was employed for the identification of the species, and the amplification products of the positive samples were subjected to digestion with the restriction enzyme HaeIII (isolated from *Haemophilus aegyptius*)[19], which was compatible with *L*. (*L*.) *infantum* (Fig 2).

The patient returned to HUMAP in January 2022 with complaints of pain in facial lesions and difficulty swallowing solid food. A physical examination revealed that the facial lesions in the right malar and bilateral periauricular regions had worsened, with the presence of granulomatous tissue on a purplish-red background.

He was admitted to the hospital for treatment of infectious diseases diagnosed in his previous hospitalization, as well as for cytomegalovirus (CMV) intestinal infection, histoplasmosis (identified from the fungal culture of a skin lesion), and COVID-19. Additionally, he was diagnosed with upper digestive hemorrhage. While hospitalized, he developed anemia and severe thrombocytopenia, with a CD4 count of 1 cell/mm^3^, requiring hemotransfusion and vasoactive drug therapy. The initial treatment consisted of 25 mg of amphotericin B deoxycholate, which was discontinued due to deterioration in renal function and replaced with liposomal amphotericin B (L-AmB). During this period, the patient underwent a 10-day course of treatment for tuberculosis, leishmaniasis, and histoplasmosis, in addition to antiretroviral therapy (ART) comprising dolutegravir 50 mg every 12 hours, tenofovir 300 mg per day, and lamivudine 300 mg per day. The patient was discharged from the hospital but was scheduled for maintenance treatment for leishmaniasis and histoplasmosis with liposomal amphotericin B (L-AmB) 200 mg once a week and follow-up with secondary prophylaxis. Subsequent to his discharge, the patient failed to adhere to the antiretroviral therapy (ART) regimen.

In March 2022, a second sample of BMA was collected from the patient during their period of hospitalization following the completion of treatment for leishmaniasis. The sample was analyzed at the LPH/INBIO/UFMS using direct and molecular parasitological diagnosis. Both methods yielded negative results. He was readmitted to HUMAP in early July 2022 due to severe anemia (hemoglobin concentration of 3.8 g/dL), profuse epistaxis, and bleeding from a skin lesion in his left nostril. However, he was discharged from the hospital three days after admission. He was readmitted to the hospital at the end of July 2022 in a significantly worsened clinical condition and passed away two days after hospitalization.

**Fig 3:**
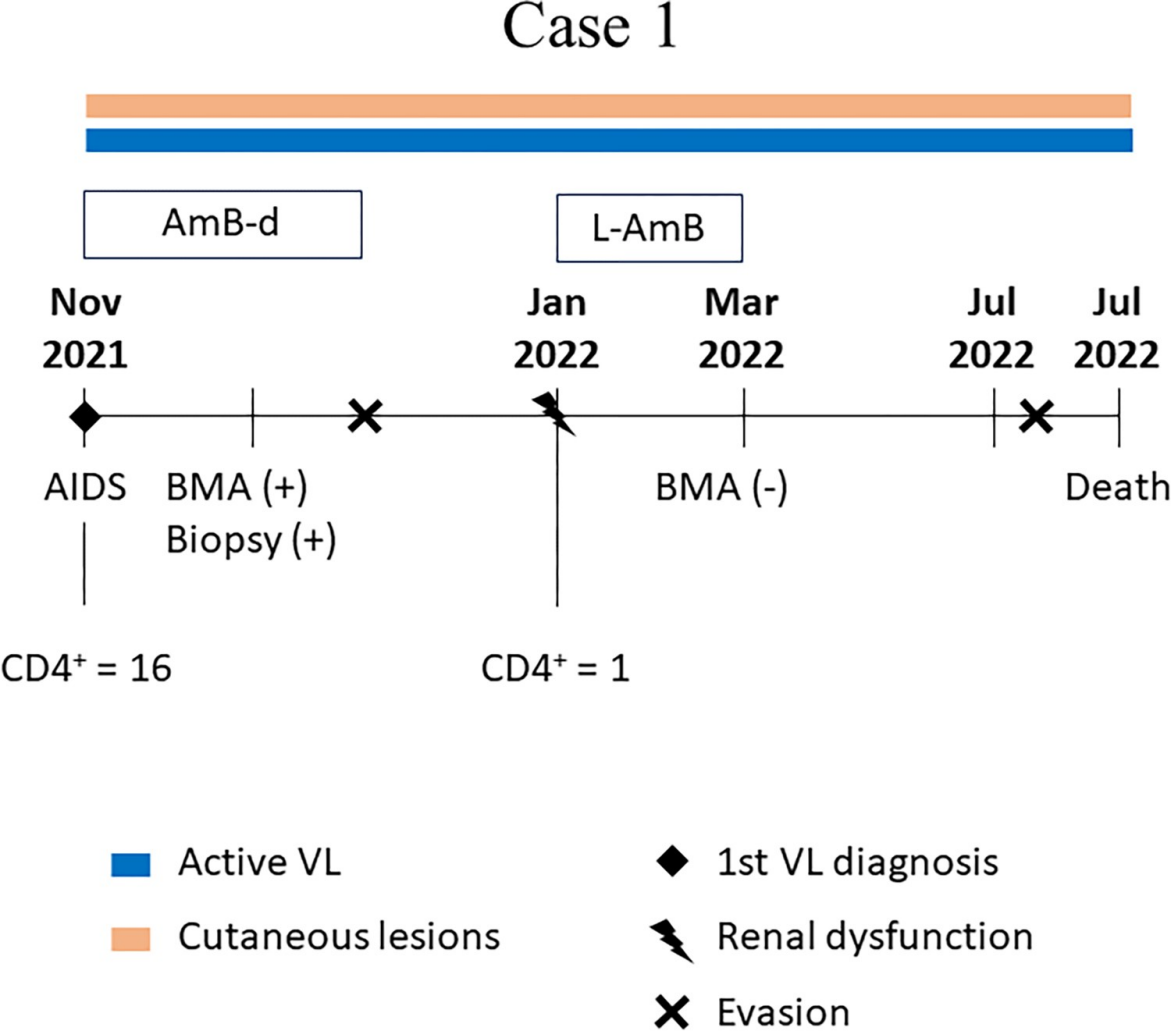
Main clinical events of Case 1. Timeline of the main events that occurred in case 1. AIDS: Acquired human immunodeficiency syndrome; L-AmB: liposomal amphotericin B; AmB-d: amphotericin B deoxycholate; BMA: bone marrow aspirate; VL: Visceral leishmaniasis; Biopsy: biopsy of skin lesions; (+) positive diagnostic; (-) negative diagnostic; CD4+: CD4 Lymphocyte count; vl: viral load.

Fig 3 presents a timeline of the principal clinical events and associated therapeutic interventions for VL.

### Case 2

A 42-year-old male patient from Campo Grande, MS, was referred to HUMAP/UFMS in May 2017 for a follow-up of AIDS, diagnosed approximately 12 years earlier (2004). The patient reported a history of alcohol use disorder but denied smoking or using illicit drugs. At the appointment, the patient was in good health and was taking ART with tenofovir, lamivudine, and efavirenz. Additionally, the patient was receiving sulfamethoxazole and azithromycin as primary prophylaxis and L-AmB (200 mg injected every 21 days) as secondary prophylaxis for VL, which was diagnosed in 2016. Laboratory tests conducted in April 2017 demonstrated a CD4 count of 93.0 cells/mm^3^ and an undetectable viral load. On physical examination, the patient was in a satisfactory general state, hemodynamically stable, and no abnormalities were observed. In June 2017, the patient exhibited symptoms of chronic diarrhea, loss of appetite, and a general decline in condition. In November 2017, the antiretroviral regimen was modified to include zidovudine, lamivudine, and efavirenz due to the development of chronic kidney disease.

Despite the administration of L-AmB every 15 days as secondary prophylaxis, the patient experienced a recurrence of VL in January 2018. Consequently, the attack treatment was reintroduced. Concomitantly, the CD4 count was 123.0 cells/mm^3^, and the viral load was undetectable. Four months later, in July 2018, the patient returned with complaints of emotional lability. Despite a diagnosis of VL and hepatosplenomegaly, the patient exhibited no apparent signs of illness upon physical examination. In September, the patient exhibited an undetectable viral load and a CD4 count of 47.0 cells/mm^3^. In February 2019, the patient experienced another relapse and received retreatment with L-AmB. In March 2019, the CD4 count was 57.0 cells/mm^3^, and the viral load was undetectable. In April 2019, the patient presented with discrete and diffuse erythematous papules and nodules on the face, anteroposterior trunk, upper limbs, and lower limbs. A skin biopsy was then performed, which revealed the presence of *Leishmania* sp. amastigotes. Concomitant severe and refractory pancytopenia was considered to be secondary to splenomegaly. In May, he underwent splenic embolization, which resulted in the development of sepsis and an episode of acute on chronic renal failure. Upon physical examination, the patient was found to be in a poor clinical state, exhibiting pallor, dehydration, and tachypnea. A leukocytosis with left deviation was identified during a blood test, and the blood cultures were found to be negative. Antimicrobial therapy with piperacillin-tazobactam and hemodialysis sessions were initiated to stabilise the patient’s condition.

The patient demonstrated a notable improvement in his overall condition during the September 2019 follow-up. Laboratory tests demonstrated a glomerular filtration rate (GFR) of 63 mL/min/1.73 m^2^, indicative of a significant recovery in kidney function. At that time, he was undergoing ART with abacavir, lamivudine, and dolutegravir. His CD4 count was 150 cells/mm^3^, and his viral load was undetectable. In November 2019, a recurrence of VL was observed, accompanied by the presence of *Leishmania* sp. amastigotes in both the BMA and skin lesion biopsy. This relapse resulted in hepatosplenomegaly and an increase in globulins, reaching 7 g/dL. Ten doses of pentamidine 150 mg were prescribed between December 2019 and January 2020. L-AmB was administered as a secondary prophylaxis every fifteen days until the CD4 count reached a level above 350 cells/mm^3^.

**Fig 4:**
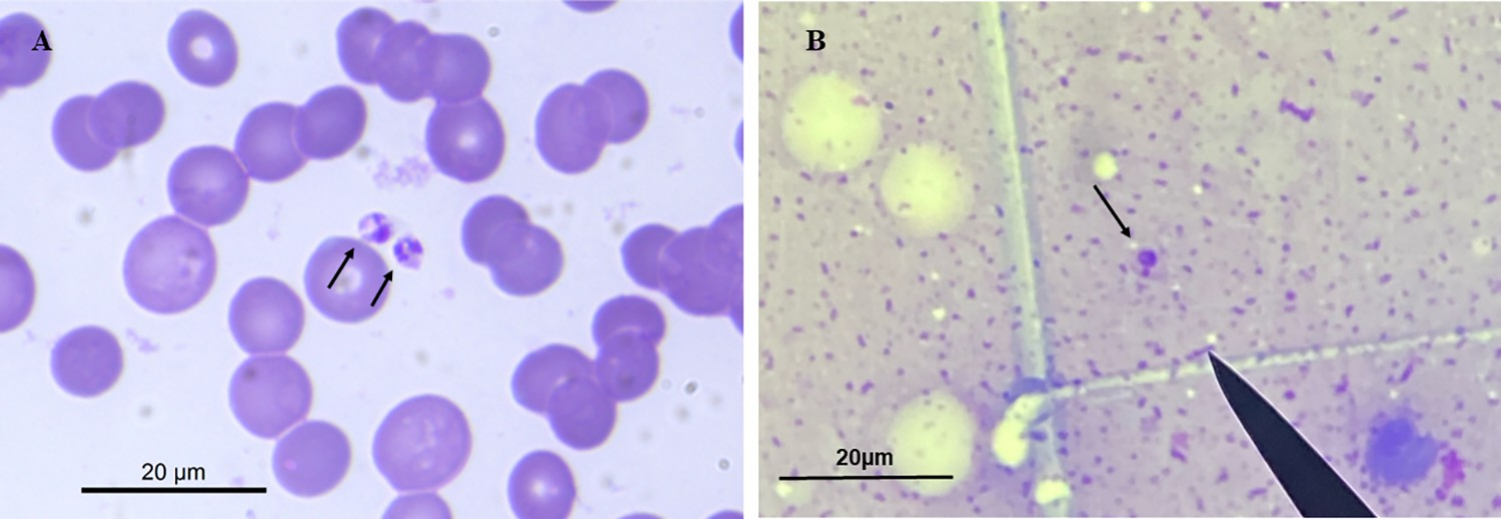
Diagnosis of leishmaniasis (Case 2): direct parasitological methods. (A) amastigote forms in bone marrow aspirate (arrows); (B) amastigote form found in a biopsy of skin lesions (arrow).

For the identification of *Leishmania* sp. species, BMA slides dated November 26, 2019 (Fig 4A), and skin lesion imprint slides dated April 9, 2019 (Fig 4B), were used. The biopsy material from the lesion was insufficient to yield a conclusive result. Nevertheless, the presence of *L*. *infantum* was confirmed in the BMA (Fig 2).

The patient returned for follow-up in February 2020 with an undetectable viral load and a CD4 count of 353.0 cells/mm^3^. By July 2020, the CD4 count had increased to 480.0 cells/mm^3^. In August 2020, the patient was discharged from secondary prophylaxis due to the favorable progress indicators and the absence of VL signs and symptoms. By November 2020, the patient’s general and nutritional condition was satisfactory, and no changes were observed in laboratory tests during this period.

The most recent appointment at HUMAP occurred in February 2022, with no reports of adverse events or patient complaints. At the present time, he is being monitored at another outpatient clinic without any symptoms related to leishmaniasis.

**Fig 5:**
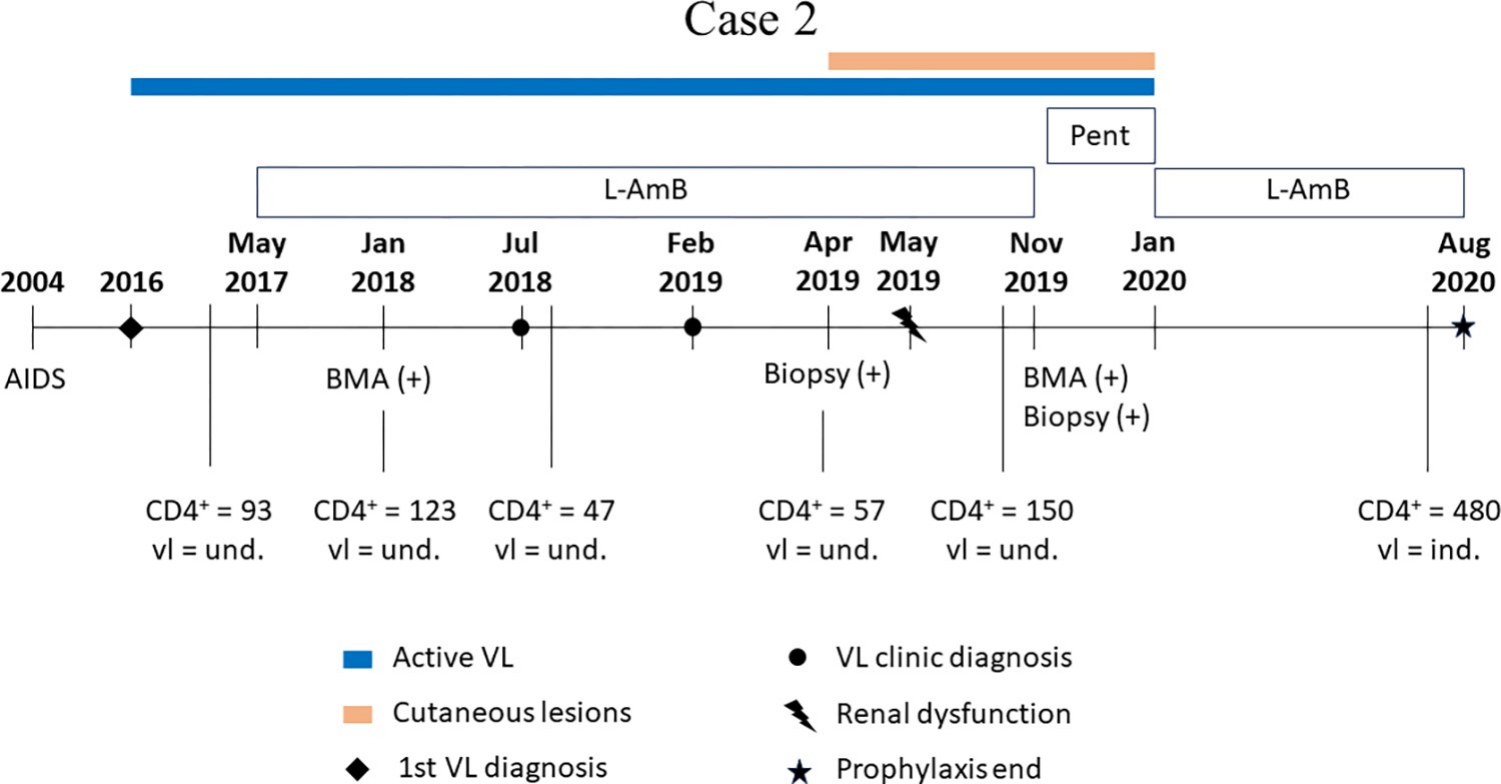
Main clinical events of Case 2. Timeline of the principal occurrences in Case 2. AIDS: Acquired human immunodeficiency syndrome; L-AmB: liposomal amphotericin B; AmB-d: amphotericin B deoxycholate; Pent: Pentamidine; BMA: bone marrow aspirate; VL: Visceral Leishmaniasis; Biopsy: biopsy of skin lesions; (+) positive diagnostic; (-) negative diagnostic; CD4+: CD4 Lymphocyte count; vl: viral load.

Fig 5 depicts the chronology of therapeutic approaches and pivotal clinical occurrences associated with VL.

### Case 3

A 46-year-old male patient, a rural worker and bricklayer’s assistant, a former smoker and drinker, and a resident of Campo Grande has been receiving follow-up care at HUMAP since 2014 due to AIDS. In 2016, he experienced an acute renal failure and was diagnosed with VL, neurotoxoplasmosis, tuberculosis, and porphyria cutanea tarda.

**Fig 6:**
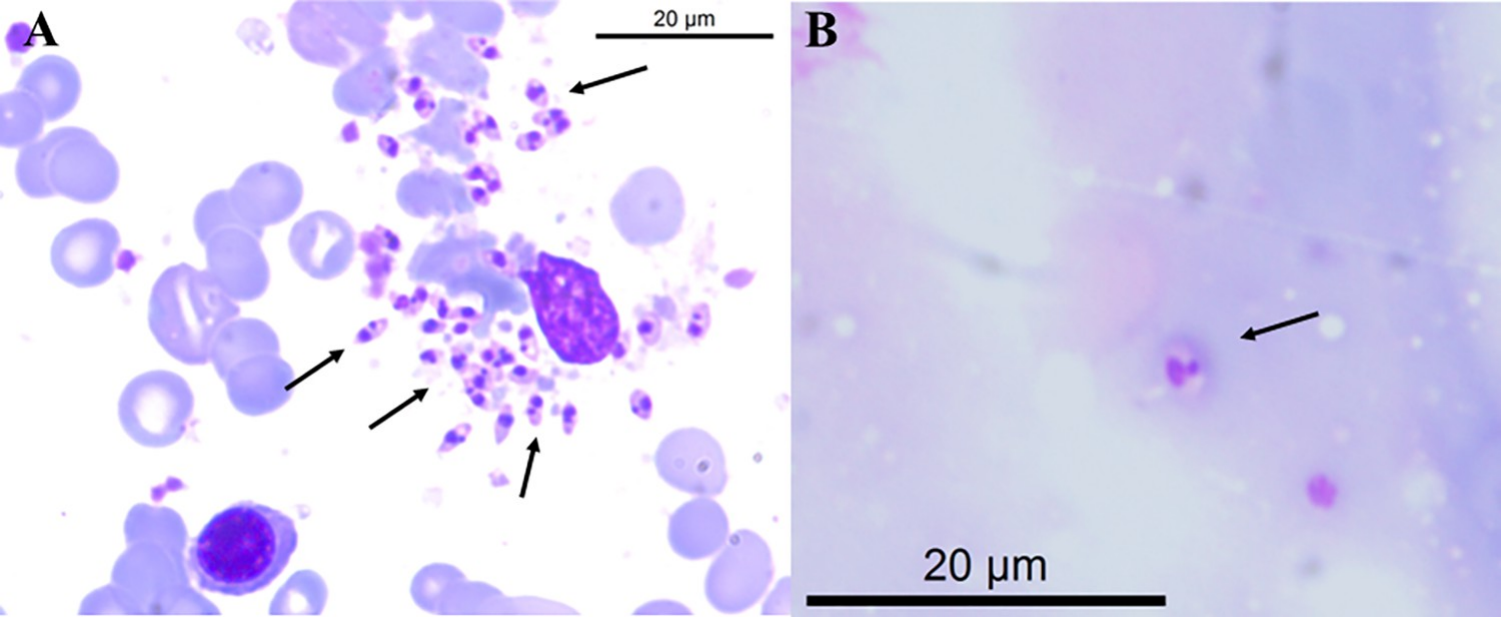
Diagnosis of leishmaniasis (Case 3): direct parasitological methods. (A) amastigote forms in bone marrow aspirate (arrows); (B) amastigote form found in a biopsy of skin lesions (arrow).

In February 2019, he was hospitalized for 13 days due to the presence of erythematous papules, bullous, crusted, and non-pruritic skin lesions on his lower limbs, which subsequently evolved to involve his upper limbs, trunk, face, and posterior cervical region (Fig 6A and 6B). At this time, the CD4 count was 121 cells/mm^3^, and the viral load was 83 copies/mm^3^. Despite the lack of improvement, in June he returned to the service complaining of spontaneous epistaxis (without triggering factors), hepatosplenomegaly, and pancytopenia. A bone marrow aspiration and biopsy of a skin lesion in the malleolar region were collected, and *Leishmania* sp. amastigotes were observed in both materials. The patient was administered liposomal amphotericin B (L-AmB) at a dose of 250 mg daily for a period of ten days, in addition to biweekly prophylaxis with the same drug.

The patient reported experiencing palpitations with a sudden onset while at rest on a monthly basis, beginning in September 2019. The patient was diagnosed with systemic arterial hypertension (SAH), hypertrophic cardiomyopathy, and pulmonary arterial hypertension. A CD4 count of 170 cells/mm^3^ with an undetectable viral load was observed in June 2020. Subsequently, the secondary prophylaxis for leishmaniasis was modified to an interval of 21 days. However, in July 2020, the dose of L-AmB was reduced to 150 mg due to alterations in renal function. Furthermore, in September 2020, the dose was reduced to 100 mg. The patient’s renal alterations persisted over the following month, necessitating his admission to the hospital for uninterrupted treatment while his renal, cardiac, and hepatic functions were closely monitored. The administration of L-AmB was gradual, beginning with a dosage of 1 mg/kg (one vial per day) for three days until a total cumulative dose of 200 mg was reached (adjusted for renal function at 3 mg/kg for a 63.5 kg patient over 10 days). In the same period, *Leishmania* spp. amastigotes were detected again due to erythematous papules persisting, as a result, a biopsy was performed at the edge of the skin lesion. The patient was discharged from the hospital 12 days after admission and then received 150 mg of secondary prophylaxis at the day hospital every 21 days. His CD4 count was 135 cells/mm^3^ with an undetectable viral load in December 2020.

BMA and biopsy of the skin lesion were performed in June 2019 and October 2020 (Fig 6A and 6B). The samples were sent to LPH/INBIO/UFMS for molecular diagnosis. As with Case 2, DNA was extracted from the scrapings of the slides, and the PCR and RFLP techniques identified *L*. (*L*.) *infantum* (Fig 2).

In February 2021, the nephrotoxicity of his medication for opportunistic infections with low functional reserve was evaluated. The treatment for leishmaniasis was modified to a thrice-weekly infusion of pentamidine with electrocardiogram (ECG) monitoring. The administration of the novel chemotherapy regimen resulted in a favorable clinical response, with an increase in the CD4 count to 223 cells/mm^3^ and a reduction in the viral load to undetectable levels.

Subsequently, a dermatologist was consulted, and a biopsy of a skin lesion on the lower abdomen was performed. The dermis exhibited a diffuse interstitial inflammatory infiltrate that contained primarily lymphocytes, histiocytes, and a few plasma cells. The presence of amastigote-like forms was observed, and an immunohistochemical analysis for *Leishmania* sp. was carried out, which yielded negative results. Consequently, secondary prophylaxis for leishmaniasis using pentamidine was scheduled to be administered biweekly.

The patient demonstrated a notable improvement in the latter half of 2022, with no new lesions, signs, or symptoms. Furthermore, the CD4 count increased to 397 cells/mm^3^, while the viral load was 45 copies/mm^3^. The patient completed the course of treatment with pentamidine following the final administration of the drug in March 2023. During a medical follow-up in July, the patient reported no fever, cough, sweating, weight loss, or other signs or symptoms of illness. Laboratory tests demonstrated a CD4 count of 353 cells/mm^3^ and an undetectable viral load. The patient was advised to return for a follow-up consultation in September.

**Fig 7:**
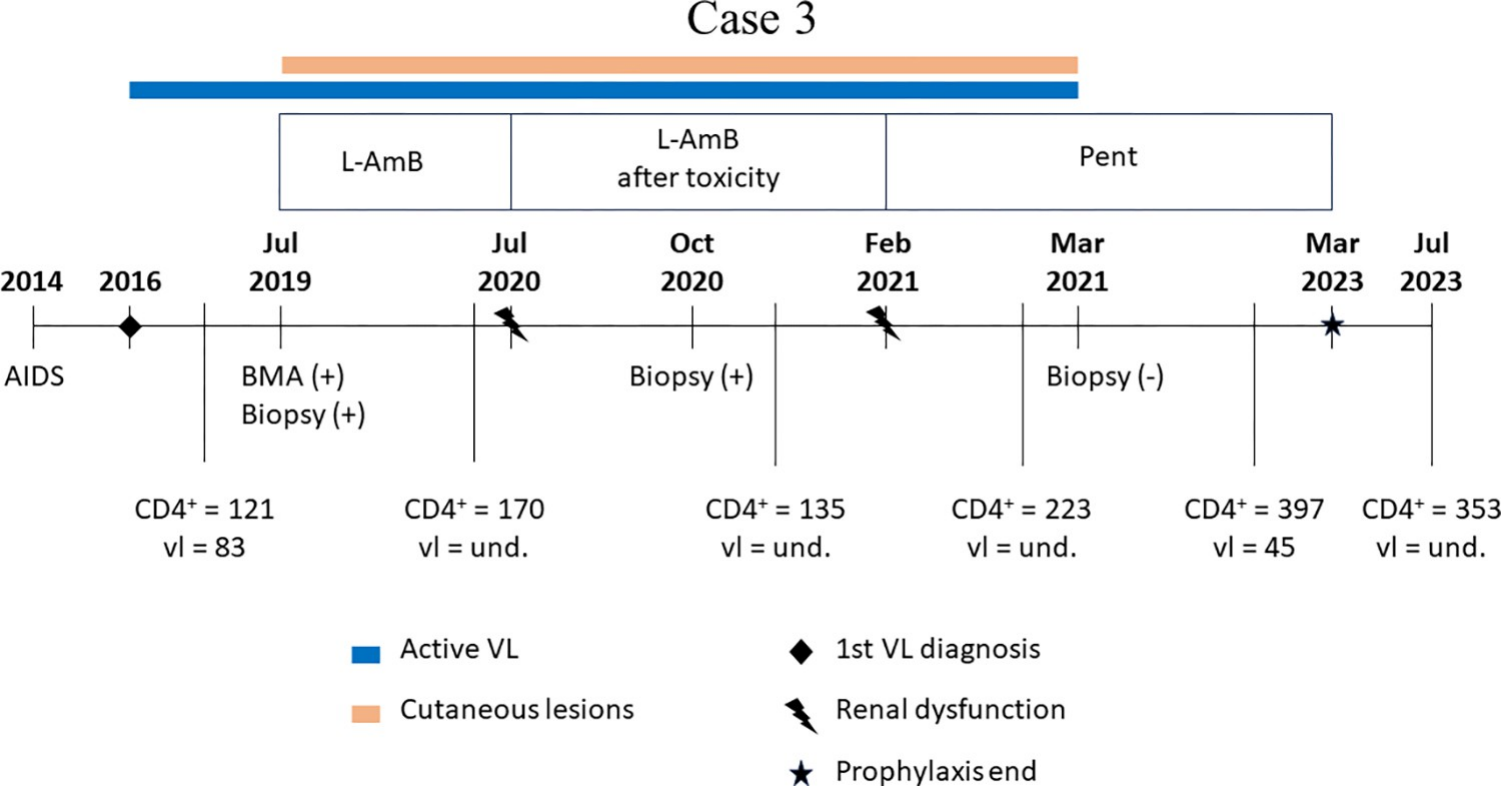
Main clinical events of Case 3. Timeline of the main events that occurred in case 3. AIDS: Acquired human immunodeficiency syndrome; L-AmB: liposomal amphotericin B; AmB-d: amphotericin B deoxycholate; Pent: Pentamidine; BMA: bone marrow aspirate; VL: Visceral Leishmaniasis; Biopsy: biopsy of skin lesions; (+) positive diagnostic; (-) negative diagnostic; CD4+: CD4 Lymphocyte count; lv: viral load.

Fig 7 illustrates the clinical events and therapeutic approach for case 3 of VL.

A brief summary of the main events of the three cases is presented in Table 1.

**Table 1:** Summary of reported cases.

Cases	1	2	3
**Direct Parasitological examination (Skin biopsy)**	Amastigote forms of *Leishmania* sp.	Amastigote forms of *Leishmania* sp.	Amastigote forms of *Leishmania* sp.
**Molecular diagnosis of Skin biopsy** **(RFLP)**	*L*. (*L*.) *infantum*	insufficient material	*L*. (*L*.) *infantum*
**Direct Parasitological examination** **(BMA)**	Amastigote forms of *Leishmania* sp.	Amastigote forms of *Leishmania* sp.	Amastigote forms of *Leishmania* sp.
**Molecular diagnosis of BMA** **(RFLP)**	*L*. (*L*.) *infantum*	*L*. (*L*.) *infantum*	*L*. (*L*.) *infantum*
**Amphotericin B deoxycholate (order of drug administration)**	Yes (1^**st**^)	No	No
**Liposomal Amphotericin B**	Yes (2^**nd**^)	Yes (1^**nd**^/3^th^)	Yes (1^**st**^)
**Pentamidine**	No	Yes (2^**nd**^)	Yes(2^**nd**^)
**Secondary Prophylaxis**	abandonment	Yes	Yes
**Relapses**	0	3	1
**Duration of follow-up**	-	5 years (2016–2020)	5 years (2019–2023)
**Outcome**	death	completed secondary prophylaxis	completed secondary prophylaxis

1^st^, 2^nd^ and 3^th^—order of administration of the medication. BMA: Bone Marrow Aspiration.

## Discussion

This study presents the first description of three cases that exhibit characteristics suggestive of para-kala-azar dermal leishmaniasis (para-KDL) in the Midwest region of Brazil. Para-KDL is characterized by the manifestation of visceral infection and skin lesions with abundant parasitic forms, and it is considered an intermediate form between PKDL and VL [9]. In immunosuppressed individuals, these manifestations are more frequent and severe [5].

The pathophysiology and immune responses in VL/HIV co-infection and para-KDL are different. In VL/HIV co-infection, the immune response is predominantly of the Th2 type, characterized by reduced levels of IL-12, IL-18, and IFN-γ. On the other hand, in para-KDL, the immune response is a combination of Th1/Th2 profiles, with production of IFN-γ and persistence of IL-10, as well as a Th17 response. While VL/HIV co-infection induces an immune response more inclined towards Th2, in para-KDL a mixture of Th1 and Th2 responses is observed, accompanied by the presence of a Th17 response [5,20,21].

Para-KDL has great epidemiological importance because human patients can serve as a reservoir for *Leishmania* spp. and play a crucial role in the anthroponotic transmission of the parasite [9,21,22].

All three case reports describe patients diagnosed with AIDS who also had active VL and concomitant skin lesions (Table 1). The clinical manifestations of VL were consistent across all cases, including fever, hepatosplenomegaly, pancytopenia, and disseminated skin lesions. Furthermore, amastigote forms were observed in both BMA and skin lesion biopsies. The presence of *L*. (*L*.) *infantum* was confirmed through RFLP analysis of the samples, providing strong support for the clinical diagnosis of para-KDL.

The available CD4 count data is described in the timeline of each case. Unfortunately, we do not have cytokine dosages to assess the systemic inflammatory response and the cutaneous inflammatory response in terms of TH1 or TH2 predominance separately. The low CD4 count suggests that a systemic TH2 response may have predominated, making it difficult to control the leishmaniasis [5].

The co-infection of HIV and *Leishmania* spp. has the potential to compromise the cellular immune response, which could in turn affect the evolution of the immune response to both agents. The observed improvement in CD4 count may have resulted from the control of *Leishmania* infection through the use of leishmanicidal drugs, while the control of skin lesions may have resulted from the improvement in CD4 count due to the use of HAART. These phenomena have been well documented in the studies of Zijlstra [5,23].

In the three cases presented here, dermal lesions cannot be classified as disseminated cutaneous leishmaniasis (DL) due to the following reasons: although the clinical characteristics of the lesions may exhibit some degree of overlap, the parasite identified in these cases was *L*. (*L*.) *infantum*, while the two species recognized as causing DL are *L*. (*Viannia*) *braziliensis* and *L*. (*L*.) *amazonensis*. In none of the cases were there reports of primary lesions before the description of multiple lesions, which could suggest hematogenous or lymphatic dissemination of the parasite, as occurs in DL. Additionally, there were no accompanying mucosal lesions, which can be present in up to 30% of patients with DL. It is notable that the prevalence of the parasite in the disseminated form is lower compared to the diffuse form. Moreover, the lesions do not correspond with the clinical presentation of diffuse cutaneous leishmaniasis (DCL), which is characterized by extensive verrucous plaques and mucosal lesions [24].

Concomitant skin involvement in AIDS-associated VL, with identification of *L*. *infantum* in the skin lesions, has been considered an atypical manifestation of VL due to severe immunosuppression [25,26]. Cases can only be characterized as para-KDL when they present clinical and immunopathogenic characteristics similar to PKDL [13], along with macropapular lesions distributed on the face, trunk, and in patients who have already started antileishmanial treatment. This suggests that specific cellular immunity has begun to be restored. However, in patients with AIDS, PKDL/para-KDL manifests in a manner distinct from that observed in non-AIDS patients. This is attributed to the profound reduction in CD4 T lymphocytes, which is accompanied by a notable prevalence of amastigotes within the lesions and a more pronounced and extensive clinical presentation [5]. The complex interaction between the immune response and the parasite gives rise to a multitude of designations that may be overlapping and even confusing. There arises, therefore, a need for the adoption of a nomenclature that allows for accurate diagnosis and proper procedures [5].

Cases 2 and 3 describe the characteristics of the chronic active visceral form of the disease, in which the continuous multiplication of parasites is observed, signs and symptoms persisting even under prophylaxis [27]. Additionally, the patients experienced relapses of VL, which is likely attributable to the immunosuppressive effects of HIV. Furthermore, immunodeficiency is associated with ART, secondary prophylaxis, and CD4 lymphocyte count [28,29].

In HIV patients, a CD4 count below 200 cells/mm^3^ is a significant prognostic factor for survival, with the potential to facilitate the development of opportunistic diseases, such as VL [30,31]. This is demonstrated by comparing the low CD4 count of the three reported patients to the onset of symptoms and the diagnosis confirmation for VL. Cases 2 and 3 demonstrated an improvement in the clinical picture upon an increase of these cells.

The clinical manifestations of HIV/VL coinfection may be atypical, involving the gastrointestinal and respiratory tracts, and kidneys, and inducing hemorrhagic phenomena (epistaxis, ecchymosis, and hematuria) [32,33]. In all cases reported, the patients exhibited chronic kidney disease. Furthermore, cases 2 and 3 exhibited evidence of cardiovascular and hepatic involvement. In Case 3, pulmonary arterial hypertension was observed, with multiple possible etiological factors (HIV infection, cardiomyopathy, chronic obstructive pulmonary disease, and chronic kidney disease).

In the Americas, the Pan American Health Organization (PAHO) recommends the use of amphotericin B for the treatment of HIV/VL coinfection, as well as the practice of secondary prophylaxis in all patients with CD4 counts below 350/mm^3^ [34].

Our service followed the recommendations of the ’Manual de Recomendações para Diagnóstico, Tratamento e Acompanhamento de Pacientes com o Coinfecção *Leishmania*-HIV’ (Manual of Recommendations for the Diagnosis, Treatment and Follow-up of Patients with *Leishmania*-HIV Coinfection) [35] during the patients’ treatment period. Accordingly, the recommended initial treatment was amphotericin B deoxycholate at a dose of 1 mg/kg/day (with a maximum daily dose of 50 mg and a total cumulative dose of 40 mg/kg/day). Alternatively, liposomal amphotericin B at a dose of 2 mg/kg/day (with a maximum daily dose of 40 mg/kg and a total cumulative dose of 40 mg/kg/day) could be considered in the event of adverse reactions or in individuals over the age of 50. As a second-line treatment, pentamidine at a dose of 4 mg/kg/day (administered in ten doses every other day) could be employed in the event of therapeutic failure with amphotericin.

In the context of the secondary prophylaxis regimen, it is recommended that the aforementioned dosages be administered every two weeks or every four weeks. The treatment plan is designed to accommodate the patient’s tolerance, taking into account factors such as the toxicity profile and potential interactions, as well as the conditions of the healthcare service [35]. In all three cases, the protocols were followed as closely as possible. However, modifications were necessary in cases 2 and 3 due to observed toxicity, with the aim of preventing further harm to the patients. Furthermore, studies have demonstrated that the administration of a combination of two drugs is an effective method for eliminating parasites in patients with PKDL and para-KDL [11,34].

In general, patients with HIV/VL have low cure rates and high mortality rates. Treatment is complex and can result in therapeutic failure, high toxicity, and drug resistance [36]. The current literature indicates that early diagnosis and treatment, as well as regular secondary prophylaxis and follow-up care provided by a multidisciplinary team, are of significant importance. This approach led to improved survival rates and even a complete clinical cure of skin lesions in Case 2. In Case 1, the patient prematurely terminated the treatment regimen, resulting in a deterioration of his condition upon his return, ultimately leading to his demise. In Cases 2 and 3, the patients were treated with a combination of drugs, initially amphotericin B, followed by pentamidine. The efficacy of this approach is evident from the favourable outcomes observed in both cases.

In Brazil, the public healthcare system includes two drugs for the treatment of VL patients: meglumine antimoniate (first-choice drug) and amphotericin B. The criteria for drug choice are related to contraindications, especially regarding the toxicity of meglumine antimoniate, which is not recommended for patients with renal or hepatic insufficiency, pregnant women and HIV patients [35]. Both treatments are conducted in a hospital setting, which may contribute to low compliance and high dropout rates.

Another factor that is worthy of consideration is the elevated expense associated with antileishmanial treatment [36]. As previously reported in Cases 2 and 3, the process from diagnosis to treatment completion can be lengthy, with Case 3 spanning over five years. The patient in Case 3 is still on secondary prophylaxis. It is important to note that all administration of drugs for VL occurs in a hospital environment, which is costly and occupies beds, supplies, and requires periodic tests to monitor drug toxicity. These costs are a significant burden on health systems and on patients themselves, who lose days of work and must pay for transportation to the health unit [37].

The report discusses cases with symptoms that may overlap with other comorbidities, including syphilis, histoplasmosis, tuberculosis, and other conditions related to the prevalent PLECT syndrome in Brazil [38,39]. This underscores the significance of para-KDL as a differential diagnosis in endemic areas, which can result in more favourable outcomes for patients.
